# Tea Grounds as a Waste Biofiller for Natural Rubber

**DOI:** 10.3390/ma17071516

**Published:** 2024-03-27

**Authors:** Aleksandra Juszkiewicz, Magdalena Maciejewska

**Affiliations:** Department of Chemistry, Institute of Polymer and Dye Technology, Lodz University of Technology, Stefanowskiego Street 16, 90-537 Lodz, Poland; 237727@edu.p.lodz.pl

**Keywords:** tea grounds, natural rubber, tea waste, biofiller, ionic liquids

## Abstract

The aim of this study was the utilization of ground tea waste (GT) left after brewing black tea as a biofiller in natural rubber (NR) composites. Ionic liquids (ILs), i.e., 1-ethyl-3-methylimidazolium lactate and 1-benzyl-3-methylimidazolium chloride, often used to extract phytochemicals from tea, were applied to improve the dispersibility of GT particles in the elastomeric matrix. The influence of GT loading and ILs on curing characteristics, crosslink density, mechanical properties, thermal stability and resistance of NR composites to thermo-oxidative aging was investigated. The amount of GT did not significantly affect curing characteristics and crosslink density of NR composites, but had serious impact on tensile properties. Applying 10 phr of GT improved the tensile strength by 40% compared to unfilled NR. Further increasing GT content worsened the tensile strength due to the agglomeration of biofiller in the elastomer matrix. ILs significantly improved the dispersion of GT particles in the elastomer and increased the crosslink density by 20% compared to the benchmark. Owing to the poor thermal stability of pure GT, it reduced the thermal stability of vulcanizates compared to unfilled NR. Above all, GT-filled NR exhibited enhanced resistance to thermo-oxidation since the aging factor increased by 25% compared to the unfilled vulcanizate.

## 1. Introduction

Tea (*Camelia sinensis*) is grown all over the world. Its largest cultivations are in China, India and several other Asian countries. Black tea is the most popular type of tea. Tea leaves are used to prepare beverages that have been among the most consumed in the world for years. The high consumption results not only from its taste attributes and refreshing effects, but also from its health benefits, which are due to the numerous active compounds contained in the tea. Phytochemicals present in tea include polyphenols (e.g., catechins, theaflavins and thearubigins), methylxanthines, alkaloids, polysaccharides, amino acids and proteins, volatile oils, vitamins, pigments and some inorganic elements [1,2].

The global consumption of tea was reported to reach about seven million tons in 2023 [3]. This led to a huge amount of tea waste in the form of tea grounds left after brewing the tea. Tea waste is classified as solid biomass waste and is mostly discharged into the environment, e.g., disposed of in landfills, or subjected to incineration or composting. Meanwhile, its utilization could bring real benefits because tea waste is light, easy to process, e.g., by grinding, and may be available at no cost.

Due to the presence of polyphenols, flavonoids and catechins, which have antioxidant properties, tea, including tea waste, has the potential to modify the properties of polymeric materials. Thus, in recent years, there has been a significant increase in interest in the use of tea as an innovative ecological and sustainable ingredient in polymer composites. Previous research shows that teas can be used as antioxidants [4,5], strengthening additives [6] and fillers in polymers [7,8,9]. This variety of applications opens the way to create innovative materials with increased functionality and ecological properties.

There are also some reports in the literature regarding the use of tea waste or phytochemicals extracted from fresh tea in elastomer composites.

Masek et al. [10] proposed using natural polyphenols extracted from green tea leaves to protect elastomers against ageing. The Sencha and Gun Powder green tea extracts were proven to have antioxidative activity that can protect the vulcanizates of ethylene–propylene copolymers (EPMs) against the action of climatic factors. The protective activity of these extracts in EPMs was comparable to that of a commercial stabilizer based on hydroxybenzophenone.

Regarding the antioxidative activity of tea polyphenols, Guo et al. [4] developed a hybrid antioxidant based on naturally extracted tea polyphenol (TP)-functionalized halloysite nanotubes (HNTs). The tubular structure of HNTs was used as a nanocontainer for the continuous release of tea polyphenol in natural rubber (NR) composites. Vulcanizates containing the hybrid antioxidant exhibited remarkable long-term thermo-oxidative aging resistance and stability due to the slow release of tea polyphenols, which very effectively captured free radicals generated during the thermo-oxidative aging process. In addition, a significant improvement in tensile properties of NR vulcanizates was achieved. The same researchers fabricated an anti-aging filler based on the green tea polyphenol supported on a silica surface [11]. The use of this TP-functionalized silica in styrene–butadiene rubber (SBR) composites significantly enhanced the resistance to thermo-oxidative and UV aging. Moreover, the tensile strength of SBR vulcanizates containing TP-functionalized silica was much better compared to the unfilled SBR and increased with the content of filler.

As far as the application of tea waste is concerned, Riyajan et al. [12] investigated the effect of tea waste used as a filler on physical properties of NR composites. However, the tensile strength of NR vulcanizate in the presence of tea waste was approximately 3.5 MPa, which was significantly lower than unfilled NR. Moreover, even the addition of a small amount of tea, i.e., 10 phr, to rubber composites filled with 30 phr carbon black significantly worsened the tensile strength and elongation at break of vulcanizates due to the agglomeration of tea waste particles. To obtain satisfactory strength parameters of vulcanizates containing carbon black and tea waste, it was necessary to modify the tea waste with methyl methacrylate monomer.

Hayeemasae et al. [9] also reported the strong ability of tea waste particles to agglomerate in the NR elastomer matrix. This resulted from the incompatibility and poor adhesion between polar tea waste and the non-polar NR elastomer. Consequently, the tensile strength of the composites dropped continuously from approximately 17 MPa to 7 MPa with increasing tea waste content in the rubber composite from 5 to 30 phr. Additionally, the elongation at break of vulcanizates was reduced from approximately 900% to 650% when the amount of tea waste increased from 5 to 30 phr. Thus, incorporation of tea waste decreased the elasticity of the NR composites. Due to the increase in the stiffness of the vulcanizates caused by the introduction of the filler, the modules at 100 and 300% relative elongation also increased with the tea waste content. For example, the modulus at 300% strain rose from 1.5 to 2 MPa when the amount of tea waste increased from 5 to 30 phr. To improve the compatibility between tea waste and NR, a silane coupling agent, i.e., bis(triethoxysilylpropyl)tetrasulfide (TESPT), was used for rubber compounds containing from 5 to 15 phr tea waste [13]. Applying TESPT increased the tensile strength of the vulcanizates filled with 10 phr tea waste by 1.5 MPa, but, for 15 phr of the filler, no significant influence of silane on the tensile strength was achieved (changes were within standard deviation), whereas elongation at break was reduced by approximately 100% compared to the vulcanizate without TESPT. Therefore, it is worth looking for new coupling or dispersing agents for tea waste to be successfully used as a filler in elastomer composites.

Therefore, in this work, we studied the possibility of utilization of ground tea waste left after brewing black tea as a biofiller in elastomer composites. The aim of this study was not only to find an effective way to manage tea grounds (GT), but also to enhance the strength parameters and poor resistance of the NR to thermo-oxidative aging. To improve the dispersibility of GT particles in the NR elastomer matrix, imidazolium ionic liquids (ILs) were employed such as 1-ethyl-3-methylimidazolium lactate (EmiLa) and 1-benzyl-3-methylimidazolium chloride (BenmiCl). Alkylimidazolium ILs, including EmiLa and BenmiCl, which are widely used for the extraction of active compounds from plants, including tea [14,15,16]. Thus, we expected that the interactions of phytochemicals present in tea waste with ILs would prevent the GT particles from agglomerating in the elastomer matrix. To our knowledge, composites filled with ground tea leaves containing ILs as dispersants have not yet been reported in the literature.

## 2. Materials and Methods

### 2.1. Materials

Natural rubber (NR), i.e., cis-1,4-polyisoprene of RSS1 type, was provided by Torimex Chemicals (Lodz, Poland) and applied as an elastomer matrix. It had a density of 0.930 g/cm^3^ and content of volatile matter of 0.56 wt%. NR composites were vulcanized using sulfur (Siarkopol, Tarnobrzeg, Poland) as a curing agent, zinc oxide (ZnO) with a specific surface area of 10 m^2^/g (Huta Bedzin, Bedzin, Poland) along with stearic acid (St.A.) (Sigma-Aldrich, Poznan, Poland) as vulcanization activators and 2-mercaptobenzothiazole (MBT) (Brenntag Polska, Kędzierzyn-Koźle, Poland) as an accelerator. Ground tea waste (GT) made from black leaf tea (country of origin: Sri Lanka, supplier: KOL-POL, Stasiowka, Poland) was applied as a waste biofiller. Additionally, ionic liquids (ILs), i.e., 1-ethyl-3-methylimidazolium lactate (EmiLa) and 1-benzyl-3-methylimidazolium chloride (BenmiCl) (Sigma-Aldrich, Poznan, Poland), were used to improve the dispersion of the waste biofiller in the elastomer matrix.

### 2.2. Preparation and Characterization of the Waste Biofiller

The black leaf tea was prepared with water immediately after boiling and left to infuse for 5 min. The tea waste material (tea grounds) was then filtered and placed in a dryer (Binder, Tuttlingen, Germany) at 70 °C for 3 days. After drying, the tea waste was ground in a PULVERISETTE 23 (Fritsch, Idar-Oberstein, Germany) ball mill operating with the following parameters: rotational rate of 300 rpm, grinding time of 15 min. After grinding, the ground tea waste was additionally sifted through a 0.25 mm sieve to separate fragments of incompletely ground stems. As a result, a waste biofiller in the form of a fine, brown powder was obtained.

The ground tea waste (GT) was characterized using thermogravimetric analysis (TGA). The measurement was carried out in two stages using a thermogravimetry/differential scanning calorimetry TGA/DSC1 (Mettler Toledo, Greifensee, Switzerland) analyzer. First, the sample of GT powder was heated in an argon atmosphere in the temperature range of 25–850 °C. Next, heating continued to 1000 °C in air. The gas flow of both gases was 50 mL/min, and the rate of heating was 10 °C/min.

### 2.3. Preparation and Characterization of NR Composites

NR composites were prepared according to the recipes presented in Table 1.

A laboratory two-roll mill (David Bridge & Co., Rochdale, UK) with roll diameter of 200 mm and roll length of 450 mm was employed to manufacture the rubber compounds. During mixing, the rotational speed of the front roll was 16 min^−1^, the friction was 1–1.2 mm and the width of the gap between rollers was in the range of 1.5–3 mm. The average temperature of the rolls during compounding was approximately 30 °C, whereas the preparation time of each rubber composite was approximately 10 min. Six rubber compounds were manufactured such as the unfilled NR composite, the rubber compounds containing from 10 to 30 phr of GT powder (10GT, 20GT and 30GT, respectively) and the NR composites containing 30 phr of GT and ILs as dispersants (30GT/EmiLa and 30GT/BenmiCl, respectively).

The curing characteristics of NR rubber compounds were recorded at 160 °C according to the ISO 6502 [17] standard procedures by using the rotorless rheometer D-RPA 3000 (MonTech, Buchen, Germany). Based on the data obtained, the optimal vulcanization time (t_90_) and the scorch time (t_02_) were determined as the time necessary for rubber compound to reach 90% and 2% of the maximum achievable torque, respectively.

Differential scanning calorimetry (DSC) measurements for NR rubber compounds were performed according to ISO 11357-1 [18] standard procedures. A DSC1 (Mettler Toledo, Greifensee, Switzerland) analyzer was employed to examine the range of vulcanization temperature and the enthalpy of vulcanization. Small pieces of rubber compounds with a mass of approximately 10 mg were placed in a sealed aluminum crucible with a capacity of 40 µL and heated from −150 °C to 250 °C in an argon atmosphere at a heating rate of 10 °C/min. Liquid nitrogen was used to cool the sample prior the measurement. The vulcanization temperature range was determined based on the T_onset_ and T_endset_ values determined for the exothermic vulcanization peak using the software STARe version 16.40 (Mettler Toledo, Greifensee, Switzerland). The same software was used to determine the vulcanization enthalpy.

Scanning electron microscopy (SEM) was employed to examine the dispersion of GT powder and other ingredients in the NR vulcanizates. SEM images of the vulcanizate fractures in liquid nitrogen were taken using a LEO 1450 SEM microscope (Carl Zeiss AG, Oberkochen, Germany). Prior to the measurements, fractures of the vulcanizates were coated with carbon to improve the quality of the SEM images.

The crosslink density was determined using the method of vulcanizate equilibrium swelling in toluene. Measurements were carried out according to the procedure described in the ISO 1817 [19] standard. The crosslink density of vulcanizates was calculated using the Flory–Rehner equation [20] and the Huggins parameter of NR–toluene interaction (*χ*) given by Equation (1), where *V_r_* is the volume of the elastomer fraction in swollen gel [21]:

(1) $$\chi =0.780+0.404V_{r},$$

A Zwick Roell 1435 (Ulm, Germany) universal testing machine was employed to examine tensile properties of NR vulcanizates. Measurements were carried out according to the ISO 37 [22] standard procedure for five dumb-bell-shaped samples of each vulcanizate with a thickness of approximately 1 mm and width of the measuring section of 4 mm.

A Zwick/Roell 3105 (Ulm, Germany) hardness tester was used to determine the Shore A hardness of NR vulcanizates. Measurements were performed for disc-shaped specimens according to the standard procedures given in ISO 868 [23].

Dynamic mechanical analysis (DMA) measurements were carried out in the tension mode by using a DMA/SDTA861e (Mettler Toledo, Greifensee, Switzerland) analyzer. During the tests, cuboidal specimens of vulcanizates with a width of 4 mm, a length of 10.5 mm and a thickness of approximately 2 mm were heated from −150 °C to 60 °C with a heating rate of 3 °C/min. The measurements were performed using a frequency of 1 Hz and a strain amplitude of 4 µm.

The resistance to thermo-oxidative aging of the NR vulcanizates was studied following the ISO 188 standard procedures [24]. To simulate the thermo-oxidative aging process, plates of the vulcanizates with a thickness of approximately 2 mm were stored in a drying chamber (Binder, Tuttlingen, Germany) at 70 °C for 14 days (336 h). Then, their tensile properties, hardness and crosslink density were determined and referred to the values obtained for non-aged vulcanizates. To quantify, the aging resistance of the vulcanizates, the aging coefficient (*A_f_*) was calculated using Equation (2) [25], where *TS* is the tensile strength of vulcanizate, and *E_b_* is the elongation at break:

(2) $$A_{f}=\frac{{\left(E_{b}\times TS\right)}_{after\;aging}}{{\left(E_{b}\times TS\right)}_{before\;aging}},$$

Thermal stability of NR vulcanizates was investigated using a thermogravimetry/differential scanning calorimetry TGA/DSC1 (Mettler Toledo, Greifensee, Switzerland) analyzer. A small piece of vulcanizates with a mass of approximately 10 mg were placed in an alumina crucible with a capacity of 70 µL and heated in the temperature range of 25–600 °C in an argon atmosphere with a gas flow of 50 mL/min and a heating rate of 20 °C/min. Then, the gas was changed to air (gas flow 50 mL/min) and heating was continued up to 800 °C with the same heating rate. The same measurement procedure was applied for studying the thermal stability of ILs.

## 3. Results and Discussion

### 3.1. Dispersion of Ground Tea Waste in the NR Composites

**Figure 1:**
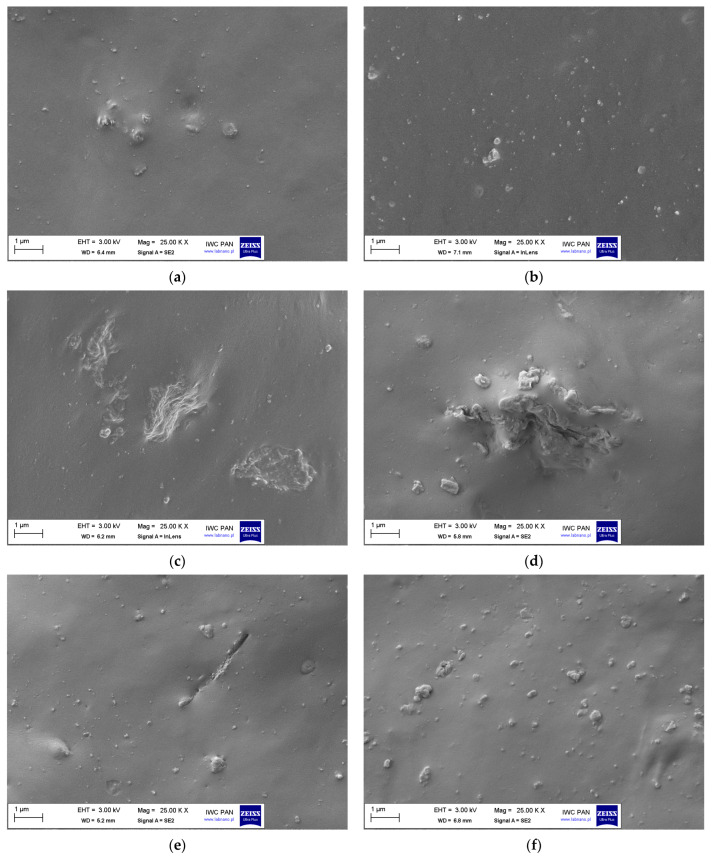
Scanning electron microscopy (SEM) images of NR composites: (**a**) Unfilled NR; (**b**) 10GT; (**c**) 20GT; (**d**) 30GT; (**e**) 30GT/EmiLa; (**f**) 30GT/BenmiCl.

Scanning electron microscopy (SEM) images of the vulcanizate fractures were taken to investigate the dispersion of ground tea waste (GT) and curatives in the NR elastomer matrix. Results are presented in Figure 1.

Regarding the unfilled NR vulcanizate, the particles of curatives were quite homogeneously dispersed in the elastomer matrix (Figure 1a). In the structure of the unfilled vulcanizate, there were only single agglomerates of particles with a size not larger than 500 nm. These agglomerates were very well wetted by the elastomer and thoroughly surrounded by the elastomer film. A similar observation was made for a vulcanizate filled with 10 phr GT (Figure 1b). Thus, 10 phr GT powder was uniformly dispersed in the elastomer. However, increasing the amount of GT to 20 phr and 30 phr resulted in a significant increase in the tendency of its particles to agglomerate in the elastomeric matrix. In the case of vulcanizate with 20 phr GT, some agglomerates of filler particles with a size of 1–2 µm were observed in the SEM image (Figure 1c), whereas, for 30 phr GT, the size of agglomerates increased to several micrometers (Figure 1d). This indicates a non-uniform dispersion of GT particles in the elastomer matrix. Since tea leaves consist mainly of lignocellulose [26], the polarity of GT weakens the interfacial interaction between the non-polar NR matrix and particles of the biofiller. Thus, for 30 phr GT, ILs were applied as potential dispersing agents. Most importantly, the use of imidazolium ILs, i.e., EmiLa and BenmiCl, significantly reduced the ability of GT particles to agglomerate and, consequently, significantly improved the dispersion of GT in the NR elastomer matrix. Despite the high GT content (30 phr), its particles were uniformly dispersed in the elastomer. SEM images of vulcanizates containing ILs (Figure 1e,f) show homogeneously distributed GT particles, forming clusters with a size of approximately 500 nm, similarly to the unfilled NR or the vulcanizate containing 10 phr of the biofiller. Thus, EmiLa and BenmiCl can be successfully used to enhance the dispersion of both GT powder and curatives in the NR composites. The improvement in the dispersion of GT particles resulting from the addition of ILs could be due to the interactions of ILs with phytochemicals present in tea. As mentioned, alkylimidazolium ILs, including EmiLa and BenmiCl, are widely used for the extraction of active compounds from plants, including tea. Many researchers have confirmed the interactions of phytochemicals present in tea with ILs through, for example, hydrogen bonds, van der Waals interactions or π–π stacking [14,15,16]. However, there is no consensus in the literature as to which of the phytochemicals present in tea preferentially interact with ILs and how exactly ILs interact with specific phytochemicals. This is difficult to determine due to the variety of organic compounds contained in tea, which additionally have various functional groups and reactivities. These groups may interact in different ways with both the cation and the anion of the IL, depending on its structure. For example, Ribeiro et al. [1] postulated the interactions of BenmiCl and EmiLa with saponins present in tea. For BenmiCl, the aromatic interactions between the aromatic group of BenmiCl and the aglycone of saponin were reported, whereas, for EmiLa, the interactions between the hydroxyl group in IL and acetyl groups in saponin were postulated. On the other hand, tea waste also contains some amino acids and proteins that possess amino groups that can interact with ILs through hydrogen bonds [14]. Therefore, determining the mechanism of interactions between ILs and phytochemicals present in tea waste that leads to the improved dispersion of GT particles in the elastomeric matrix requires further systematic research.

### 3.2. Effect of Ground Tea Waste on the Vulcanization of NR Composites

Rheometric measurements and differential scanning calorimetry (DSC) were used to investigate the influence of GT and dispersants on the vulcanization of NR composites. The cure characteristics of NR compounds at 160 °C are presented in Table 2.

Considering the standard deviation of the obtained data, the use of GT as a filler and ILs as dispersants did not significantly affect the minimum torque (S_min_) during the vulcanization. Since S_min_ correlates with the viscosity of the uncured rubber compound, it could be concluded that both GT and ILs had no considerable effect on the viscosity of uncured NR composites, which is important for their processing.

The increase in torque (ΔS) during vulcanization results from both the increase in rubber compound stiffness caused by crosslinking and the hydrodynamic effect of the filler. Hence, it correlates with the degree of rubber crosslinking and the activity of the filler. The use of GT resulted in an increase in ΔS compared to the unfilled NR. Moreover, ΔS increased with the amount of GT in the vulcanizate due to the hydrodynamic effect of GT, resulting from the increase in composite stiffness caused by the introduction of an increasing amount of the stiff filler phase. Since the presence and amount of GT did not significantly affect the crosslink density (ν_t_) of the vulcanizates (Table 2), it was concluded that the increase in ΔS compared to the unfilled NR was mainly due to the hydrodynamic effect of GT. Applying ILs resulted in an additional increase in ΔS as compared to 30GT. This resulted from the increased crosslinking degree of the elastomers containing ILs, as evidenced by the values of ν_t_. Thus, ILs enhanced the efficiency of vulcanization. This could be due to the improvement in the dispersion degree of the components of NR composites, including curatives, as evidenced by the SEM images discussed above.

Addition of GT and dispersants had no influence on the scorch time (t_02_) of NR compounds and, thus, on the safety of their processing. Considering the standard deviation of the obtained data, the use of GT and ILs did not significantly affect the optimal vulcanization time (t_90_) of rubber compounds compared to the unfilled NR. The t_90_ of the tested NR compounds was approximately 2 min, regardless of their composition.

**Figure 2:**
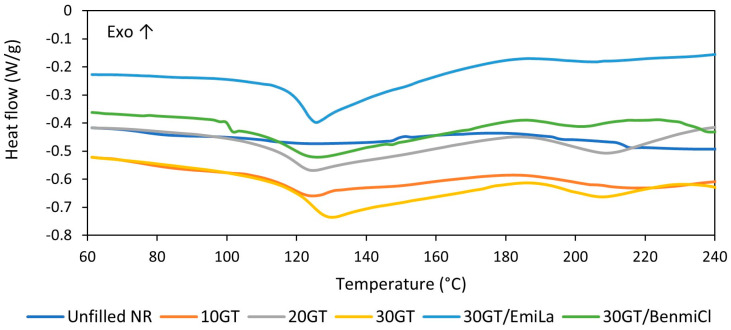
Differential scanning calorimetry (DSC) curves for vulcanization of NR composites filled with ground tea waste.

Having explored the influence of GT and ILs on the cure characteristics of NR composites, we then examined their effect on the temperature and enthalpy of vulcanization using DSC. Results are summarized in Table 3, whereas DSC curves for NR composites are shown in Figure 2.

**Figure 3:**
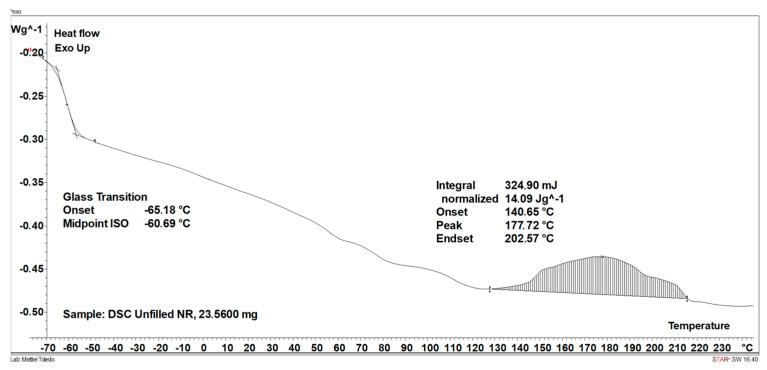
DSC curve for vulcanization of the unfilled NR composite—determination of vulcanization temperature and enthalpy by the STARe software.

**Figure 4:**
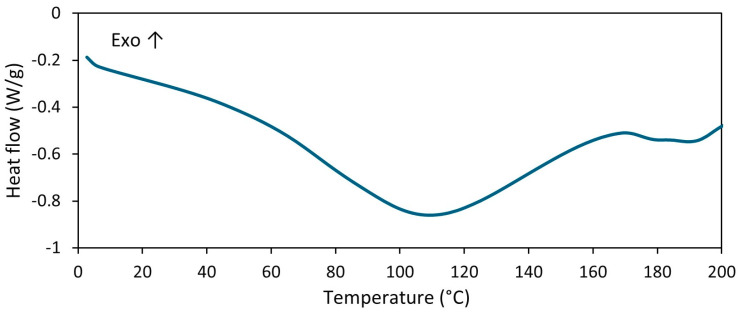
DSC curve for pure GT powder.

Figure 3 shows an example of the way in which the range of vulcanization temperature and vulcanization enthalpy for the unfilled NR composite were determined using the STARe version 16.40 software. In the same way, the vulcanization temperature and enthalpy were determined for other rubber compounds. Vulcanization of rubber compounds is an exothermic process; thus it can be, identified as an exothermic peak on a DSC curve. By integrating this peak, the amount of heat released in the vulcanization can be determined, i.e., the enthalpy of vulcanization, as well as the temperatures at which the vulcanization process begins and ends (T_onset_ and T_endset_, respectively). Regarding the DSC curve of the unfilled NR, it was noticed that vulcanization proceeded in a temperature range of 140–210 °C with an enthalpy of approximately 14 J/g. Vulcanization of NR compounds filled with GT started at a temperature approximately 14 °C lower compared to the unfilled NR and proceeded with a higher enthalpy. The possibility of reducing the vulcanization temperature is, therefore, an additional benefit of using GT as a filler for NR compounds. According to Hayeemasae et al. [9], the positive influence of tea waste on vulcanization could be due to the amine content, which increases the alkalinity of the rubber compound and, consequently, facilitates crosslinking. The amount of GT and the addition of ILs as dispersants did not significantly affect the temperature of vulcanization. However, the addition of BenmiCl slightly changed the course of the DSC curve in the vulcanization temperature range. In the case of rubber compound 30GT/BenmiCl, the second fuzzy peak following the vulcanization peak occurred in the temperature range of 203–235 °C. Thus, some post-curing reactions may be expected at this step, e.g., cis–trans isomerization, oxidative degradation of cis-1,4-isoprene units with oxygen radicals, formation of pendant sulfide groups (or scission of elastomer chains) and formation of volatile thermal degradation products [27]. Similar influence of ILs with halide anions on curing reactions was noted for styrene–butadiene and ethylene–propylene-diene elastomer composites [28,29,30]. On the other hand, for rubber compounds filled with GT, a change in the position of the baseline in the endothermic direction at a temperature of approximately 130 °C can be observed in the DSC curves before the appearance of the exothermic vulcanization peak. This change was higher with increased GT content in the rubber composite. This resulted from the desorption of bulk or bound water contained in the GT powder. This is confirmed by the DSC curve for pure GT powder, which showed a broad endothermic peak of water desorption in the temperature range of 30–160 °C (Figure 4).

Most importantly, neither the addition of GT nor the addition of imidazolium ILs had a harmful effect on the vulcanization temperature compared to the unfilled NR composite.

Considering the influence of GT and ILs on the curing characteristics and vulcanization temperature, it can be concluded that both of these components did not have a negative impact on the processing of NR composites, which is important for technological reasons.

### 3.3. Effect of Ground Tea Waste on the Mechanical Properties of NR Composites

In the next step of the research, the impact of the biowaste GT and ILs on mechanical properties and hardness of NR vulcanizates was established. Results are presented in Table 4.

Mechanical properties of NR vulcanizates strongly depended on the amount of GT used. Regarding the stress at 300% elongation (SE_300_), vulcanizates filled with GT exhibited higher SE_300_ compared to the unfilled NR. Moreover, SE_300_ increased with the content of GT in the vulcanizate. It was due to the increase in the stiffness of vulcanizates resulting from the addition of filler. Consequently, vulcanizates filled with GT showed lower elongation at break (E_b_) compared to the unfilled benchmark, with E_b_ significantly decreasing with an increase in the amount of filler. ILs did not significantly affect the SE_300_ compared to vulcanizate 30GT. On the other hand, vulcanizates with ILs exhibited approximately 100% lower E_b_ compared to 30GT. The decreased elasticity of vulcanizates containing ILs resulted from their higher crosslink density as compared to 30GT.

It should be noted that applying 10 phr GT significantly improved the tensile strength (TS) of NR vulcanizate. TS of vulcanizate filled with 10 phr GT was 4.4 MPa higher than the unfilled NR, so the improvement of TS was approximately 40%. Moreover, TS of NR vulcanizate filled with 10 phr GT was much better than the TS of vulcanizates containing 10–30 phr of commercially used fillers, e.g., silica or talc [31]. Unfortunately, TS decreased significantly with increasing GT amount in the composite. It was due to the ability of the filler to agglomerate in the elastomer matrix, as evidenced by the SEM images discussed earlier. Thus, TS of the vulcanizate filled with 20 phr GT (20GT) was approximately 3.4 MPa lower compared to 10GT, but it was still slightly better than that of the unfilled NR. However, increasing the GT content to 30 phr resulted in a deterioration of TS compared to the unfilled vulcanizate. Although both ILs improved the dispersion of the GT particles in the elastomer, they did not result in an improvement in TS compared to sample 30GT. This may be due to the increase in the crosslink density of the vulcanizates caused by the addition of ILs. It is known that TS increases with increasing ν_t_ to a certain critical value of ν_t_, beyond which the vulcanizate becomes over-crosslinked and, consequently, more brittle than the vulcanizate crosslinked to the optimal degree [32].

The use of GT increased the hardness of the vulcanizates by 3–4 Shore A compared to the unfilled one, and the GT content did not have a significant impact on the hardness. Surprisingly, the use of ILs resulted in an almost two-fold reduction in the hardness of vulcanizates compared to 30GT. The hardness of vulcanizates containing ILs was in the range of 11–15 Shore A. Moreover, the vulcanizates containing ILs had a slightly porous structure. It was noticed that, during the vulcanization of rubber compounds containing ILs, gas was released due to the interaction between ILs and phytochemicals present in tea [33], which caused the vulcanizate to immediately expand after removal from the mold and then return to a size slightly larger than the size of the mold cavity. The formation of such a vulcanizate structure could also be the second reason for the lack of a positive effect of ILs on the TS of vulcanizates despite the improvement in the degree of GT dispersion in the elastomer.

### 3.4. Effect of Ground Tea Waste on the Dynamic Mechanical Properties of NR Composites

**Figure 5:**
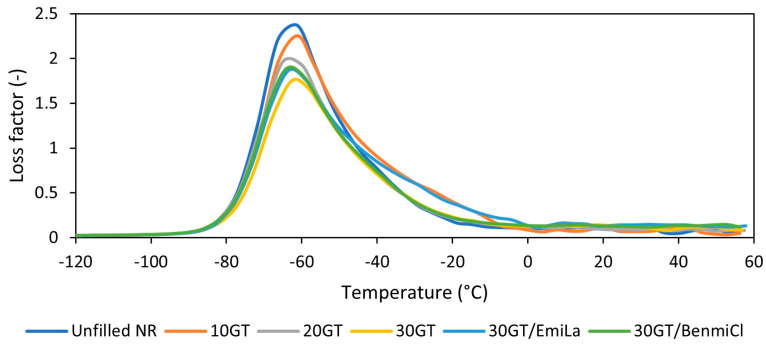
Loss factor (tan δ) curves versus temperature of NR composites filled with ground tea waste.

Having investigated mechanical properties in static conditions, we then employed dynamic mechanical analysis (DMA) to study the influence of GT biofiller and ILs on the mechanical properties of NR vulcanizates in dynamic conditions and their ability for dampen vibrations. DMA curves of the vulcanizates are plotted in Figure 5, and the results are summarized in Table 5.

The mechanical loss factor (tan δ) curves were collected as a function of temperature to determine the glass transition temperature (T_g_) of NR in the studied composites. The T_g_ was determined as the temperature of the maximum of the tan δ peak present in the DMA curves. The application of GT as a filler or ILs as dispersants had no significant effect on T_g_ of NR elastomer, which was approximately −62 °C for most vulcanizates. On the other hand, the addition GT slightly decreased the value of tan δ at T_g_ compared to the unfilled NR. Moreover, tan δ at T_g_ decreased with increasing GT content in the vulcanizate. This was due to the reduced elasticity of the vulcanizates with increasing content of the filler, as evidenced by the values of SE_300_ and E_b_ discussed earlier. However, in the rubbery elastic region, so, in the temperature range of 25–50 °C, the values of tan δ were slightly higher for the vulcanizates filled with GT. The tan δ, as the ratio of the loss modulus to the storage modulus, is a measure of the energy dissipation of a material [34,35]. The greater the tan δ, the better the material’s ability to dampen vibrations. Thus, the use of GT slightly improved the ability of NR composites to dampen vibrations in the rubbery elastic region compared to the unfilled NR. ILs did not significantly affect the values of tan δ compared to 30GT; thus, their addition should not deteriorate the damping abilities of NR vulcanizates.

### 3.5. Effect of Ground Tea Waste on the Thermo-Oxidative Aging Resistance of NR Composites

**Figure 6:**
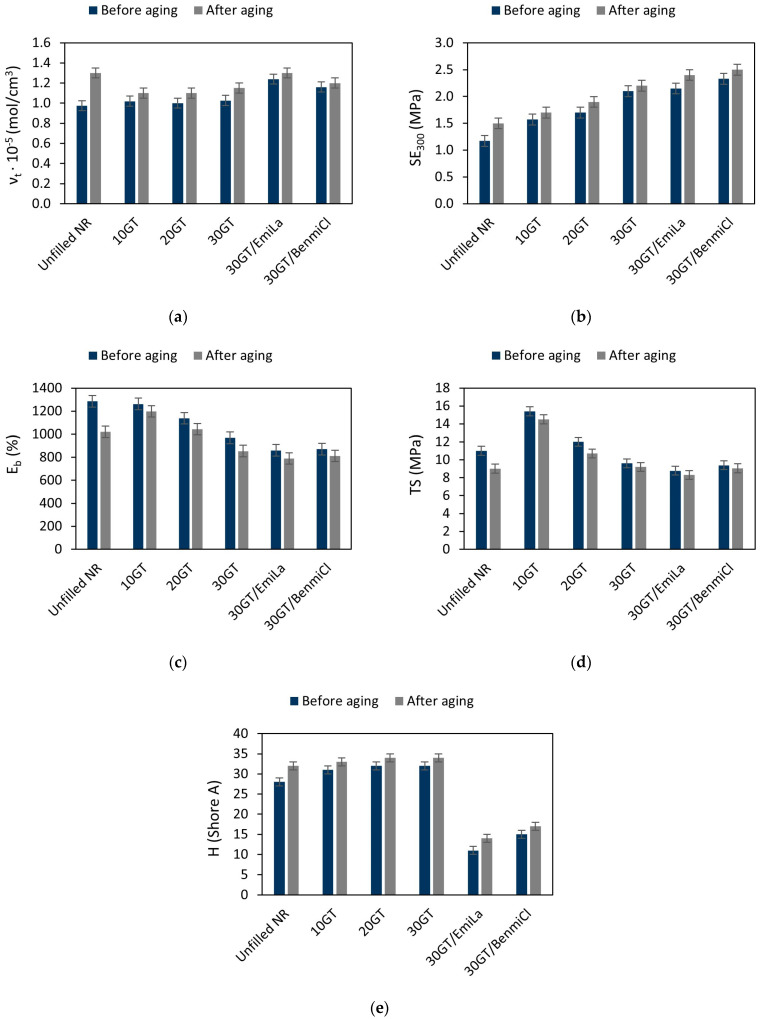
Effect of thermo-oxidative aging on the properties of NR composites filled with ground tea waste: (**a**) crosslink density; (**b**) stress at 300% relative elongation; (**c**) elongation at break; (**d**) tensile strength; (**e**) hardness.

Black tea is commonly known to contain polyphenol compounds, such as theaflavins and thearubigins formed during fermentation by the oxidation and polymerization of catechins, the major components in tea leaves [36,37]. On the other hand, phenolic compounds, including natural polyphenols, are one group of widely studied compounds that are useful as antioxidants for polymers [38]. Thus, it was reasonable to study the influence of GT biofiller on the resistance of NR composites to thermo-oxidative aging. The resistance of NR vulcanizates filled with GT to prolonged thermo-oxidation was determined based on the changes in their crosslink density, tensile properties, and hardness. Results are presented in Figure 6.

Subjecting the vulcanizates to prolonged thermo-oxidation (70 °C, 14 days) caused a significant increase in the ν_t_ of the unfilled NR as compared to the non-aged composite. In the case of vulcanizates filled with GT, an increase in ν_t_ after thermo-oxidative aging was also observed, but it was much smaller than for the unfilled vulcanizate. Consequently, vulcanizates, especially the unfilled one, exhibited higher SE_300_ after thermo-oxidative aging compared to non-aged vulcanizates. Moreover, the increase in ν_t_ caused the reduction of vulcanizates E_b_ due to thermo-oxidative aging. This is particularly visible for the unfilled vulcanizate, whose E_b_ after aging decreased by approximately 265% compared to the non-aged vulcanizate. The reduction of E_b_ was accompanied by a significant deterioration of the TS of the unfilled vulcanizate compared to non-aged material. For vulcanizates filled with GT, only a slight deterioration of TS was observed due to thermo-oxidative aging. As expected, the increase in crosslink density due to aging resulted in an increase in the hardness of the vulcanizates by 2–4 Shore A, with the highest increase in hardness achieved for the unfilled vulcanizate.

It was, therefore, noticed that thermo-oxidative aging caused the greatest changes in properties in the case of the unfilled vulcanizate. Thus, it can be expected that it shows the lowest resistance to thermo-oxidative aging. To confirm this quantitatively, the aging coefficient A_f_ was determined based on the change in tensile properties, i.e., TS and E_b_, of the vulcanizates during the aging process. Results are presented in Table 6.

A_f_ was determined as the ratio of the product of TS and E_b_ of the vulcanizate after aging to the corresponding values before aging. Therefore, the closer A_f_ is to one, the smaller the changes in the mechanical properties of the material because of aging and, therefore, the better its resistance to aging. Regardless of the filler amount, vulcanizates filled with GT were characterized by a significantly higher A_f_ compared to the unfilled NR. Thus, the use of GT improved the resistance to thermo-oxidative aging of NR composites. It was probably due to the antioxidant effect of phytochemicals, i.e., polyphenols, contained in tea. Guo et al. confirmed that tea polyphenols can effectively enhance the thermo-oxidative stability of elastomers [20]. Moreover, ILs also had a beneficial influence on the resistance of NR vulcanizates to thermo-oxidative aging. Vulcanizates with ILs showed a slightly higher A_f_ compared to the vulcanizate without ILs, i.e., 30GT. Hence, alkylimidazolium ILs such as EmiLa and BenmiCl can be successfully used as GT dispersants in NR elastomer matrix, without any harmful effect on the resistance of the composites to thermo-oxidative aging.

### 3.6. Effect of Ground Tea Waste on the Thermal Stability of NR Composites

**Figure 7:**
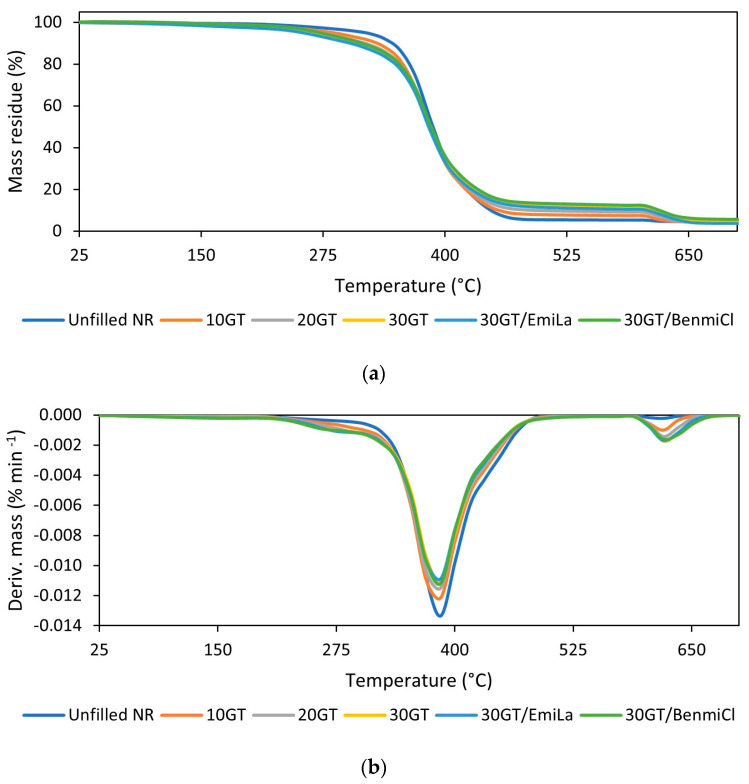
Thermogravimetric (TG) and derivative thermogravimetric (DTG) curves of the NR vulcanizates filled with ground tea waste: (**a**) TG curves; (**b**) DTG curves.

In the last step of the research, the influence of GT used as a waste filler of NR composites on their thermal stability was explored using thermogravimetry (TG). TG and DTG curves of NR vulcanizates are presented in Figure 7. TG results are summarized in Table 7.

**Figure 8:**
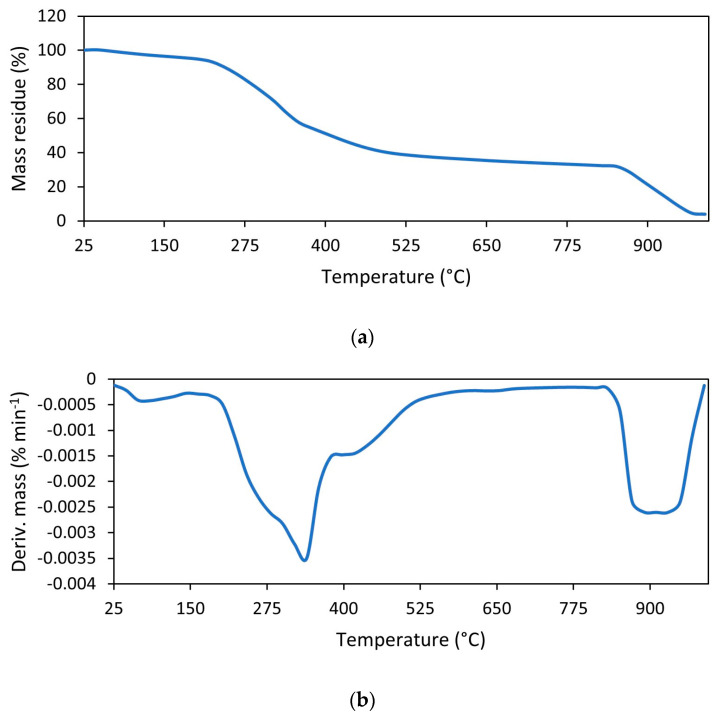
TG and DTG curve for ground tea waste: (**a**) TG; (**b**) DTG.

The onset decomposition temperature of NR vulcanizates was determined as the temperature at which the sample mass decreased by 5% in relation to its initial mass (T_5%_). The results of TG analysis presented in Table 7 show that the use of GT and its amount significantly affected the thermal stability of NR composites. The T_5%_ of the vulcanizates filled with GT was 32–52 °C lower than the unfilled NR and decreased with the increase in GT content in the vulcanizate. Thus, GT reduced the thermal stability of NR composites compared to the unfilled vulcanizate. This resulted from much lower thermal stability of pure GT powder compared to NR. When analyzing TG results presented in Figure 8 and Table 8, it was noticed that T_5%_ of pure GT powder was approximately 195 °C, i.e., significantly lower than that of the NR without addition of GT (T_5%_ of 317 °C). Regarding the thermal decomposition of pure GT, during heating in the temperature range of 25–160 °C, GT lost bulk water and bound water, which corresponded to a weight loss of approximately 4% [39]. Next, the pyrolysis of organic compounds present in tea, i.e., polyphenols, alkaloids, amino acids, proteins and pigments, occurred in the temperature range of 160–850 °C, with a mass loss of approximately 64% [2]. Finally, the combustion of pyrolysis residues took place after changing the measurement atmosphere to air at a temperature above 850 °C. The low stability of pure GT compared to NR was also confirmed by the lower T_DTG_, so the temperature at which the thermal decomposition occurred proceeded with the highest rate. T_DTG_ of pure GT was approximately 60 °C lower than unfilled NR.

**Figure 9:**
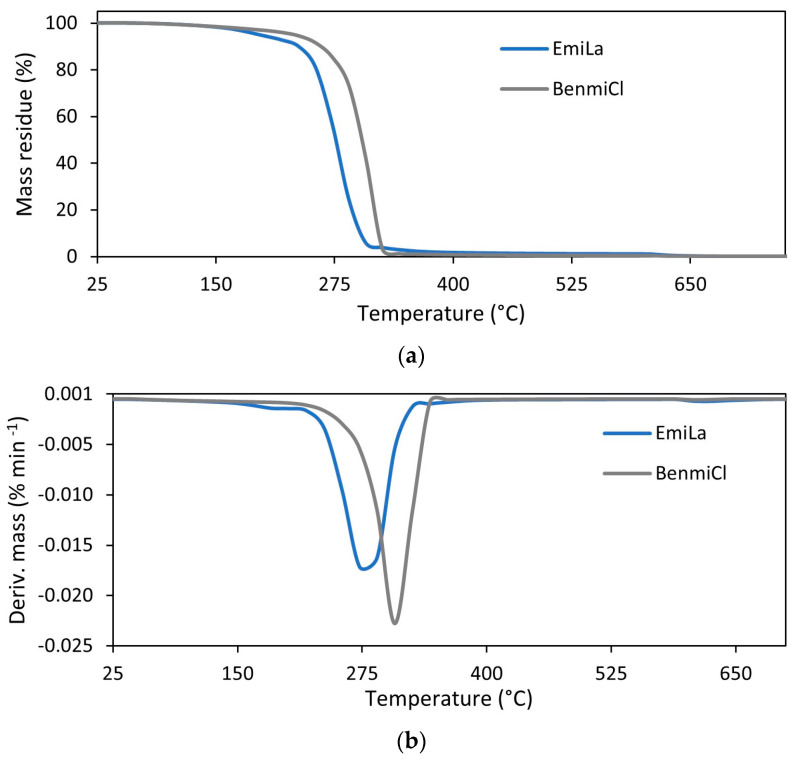
TG and DTG curves for ILs: (**a**) TG; (**b**) DTG.

The influence of ILs on the thermal stability of vulcanizates depended on the structure of IL used. This is because the thermal stability of the ILs themselves depends strongly on their structure, i.e., the types of cation and anion [40,41]. Regarding the thermal stability of pure ILs (Figure 9, Table 9), BenmiCl exhibited significantly higher thermal stability than EmiLa, as evidenced by the higher values of T_5%_ and T_DTG_, respectively. Moreover, T_5%_ and T_DTG_ of EmiLa were lower compared to pure GT powder. Consequently, addition of EmiLa reduced the T_5%_ of the NR composite filled with 30 phr GT by 8 °C compared to the vulcanizate without EmiLa. On the other hand, thermal stability of BenmiCl was higher than pure GT powder; therefore, BenmiCl, in contrast to EmiLa, slightly improved the thermal stability of the vulcanizate filled with 30 phr GT.

It should be noted that both GT and ILs had no significant effect on the T_DTG_ of NR vulcanizates, i.e., the temperature at which thermal decomposition occurred most rapidly. For most vulcanizates, the T_DTG_ was approximately 396 °C.

The mass loss during the thermal decomposition corresponded to the composition of NR composites. The first mass loss determined in argon in the temperature range of 25–600 °C was due to the pyrolysis of organic ingredients such as NR, GT, stearin, vulcanization accelerators and ILs (in the case of vulcanizates that contained them). The second mass loss determined in air in the temperature range of 600–800 °C occurred as a result of combustion of the residue after pyrolysis of organic ingredients. In turn, the residue after the thermal decomposition of the vulcanizates at 800 °C consisted of ash and zinc oxide, which was used as a vulcanization activator.

## 4. Conclusions

Ground tea waste left after brewing tea can be successfully applied as a waste plant biofiller in NR composites, as an alternative to common mineral fillers, e.g., talc or silica. Taking into account the amount of grounds produced as waste after brewing black tea, their use as an NR biofiller is simple, cheap and, therefore, strongly justified.

Regardless of the amount of filler, GT did not significantly affect the crosslink density and optimal vulcanization time of NR composites compared to the unfilled NR. On the other hand, due to the amine content, GT allowed a great reduction in the temperature at which vulcanization began.

The optimal GT content for the mechanical properties of vulcanizates was 10 phr, which allowed for a 40% improvement in tensile strength compared to unfilled NR. Increasing the GT content to 30 phr resulted in a deterioration of mechanical properties due to the agglomeration of GT particles in the elastomer matrix. Imidazolium ILs were successfully adopted to ameliorate the dispersion of GT in the NR elastomer matrix. Despite the improvement in the degree of filler dispersion, the use of ILs did not increase the strength parameters of the vulcanizates due to their high crosslink density and the microporous structure formed during the vulcanization of the rubber compounds.

The use of GT slightly improved the ability to dampen vibrations in the rubbery elastic region compared to the unfilled NR vulcanizate. However, due to the lower thermal stability of pure GT compared to NR, its application worsened the thermal stability of NR composites.

On the other hand, vulcanizates filled with GT showed significantly enhanced resistance to thermo-oxidative aging as compared to the unfilled NR, which is an additional benefit of using this biowaste as a filler in NR composites.

## Figures and Tables

**Table 1:** General recipes of NR composites used in this study, phr (parts per hundred of rubber).

Composite	Unfilled NR	10GT	20GT	30GT	30GT/EmiLa	30GT/BenmiCl
NR	100	100	100	100	100	100
ZnO	5	5	5	5	5	5
St.A.	1	1	1	1	-	-
Sulfur	2	2	2	2	2	2
MBT	2	2	2	2	2	2
GT	-	10	20	30	30	30
EmiLa	-	-	-	-	3	-
BenmiCl	-	-	-	-	-	3

**Table 2:** Cure characteristics at 160 °C and crosslink density of NR composites filled with ground tea waste (S_min_—minimum torque, ΔS—torque increment during vulcanization, t_02_—scorch time, t_90_—optimal vulcanization time, ν_t_—crosslink density; SD: S_min_ ± 0.2 dNm, ΔS ± 0.5 dNm; t_02_ ± 0.2 min; t_90_ ± 0.5 min; ν_t_ ± 0.05 × 10^−5^ mol/cm^3^).

NR Composite	S_min_ (dNm)	∆S (dNm)	t_02_ (min)	t_90_ (min)	ν_t_ × 10^−5^ (mol/cm^3^)
Unfilled NR	0.5	4.8	0.5	1.6	0.98
10GT	0.5	5.6	0.5	1.9	1.02
20GT	0.4	6.2	0.5	1.9	1.00
30GT	0.4	6.7	0.6	2.2	1.03
30GT/EmiLa	0.3	8.0	0.5	1.9	1.24
30GT/BenmiCl	0.3	7.2	0.5	2.0	1.16

**Table 3:** Vulcanization temperature (T_vul_) and enthalpy (ΔH_vul_) of NR composites determined by DSC (SD: T_vul_ ± 2 °C; ΔH_vul_ ± 1.0 J/g).

NR Composite	T_vul_ (°C)	−∆H_vul_ (J/g)
Unfilled NR	140–202	14.1
10GT	126–208	17.5
20GT	126–206	21.1
30GT	127–204	18.9
30GT/EmiLa	126–205	24.3
30GT/BenmiCl	126–203	21.6

**Table 4:** Mechanical properties of NR vulcanizates filled with ground tea waste (SE_300_—stress at a relative elongation of 300%; TS—tensile strength; E_b_—elongation at break; H—hardness).

NR Composite	SE_300_ (MPa)	TS (MPa)	E_b_ (%)	H (Shore A)
Unfilled NR	1.2 ± 0.1	11.0 ± 0.4	1285 ± 55	28 ± 1
10GT	1.6 ± 0.1	15.4 ± 0.3	1222 ± 50	31 ± 1
20GT	1.7 ± 0.1	12.0 ± 0.4	1137 ± 58	32 ± 1
30GT	2.1 ± 0.1	9.6 ± 0.3	969 ± 32	32 ± 1
30GT/EmiLa	2.2 ± 0.1	8.8 ± 0.7	860 ± 34	11 ± 1
30GT/BenmiCl	2.3 ± 0.1	9.4 ± 0.6	870 ± 31	15 ± 1

**Table 5:** Glass transition temperature (T_g_) and loss factor (tan δ) of NR vulcanizates filled with ground tea waste, determined by DMA.

NR Composite	T_g_ (°C)	tan δ_Tg_ (-)	tan δ_25°C_ (-)	tan δ_50°C_ (-)
Unfilled NR	−62 ± 1	2.4 ± 0.1	0.07 ± 0.02	0.06 ± 0.01
10GT	−62 ± 1	2.2 ± 0.1	0.07 ± 0.02	0.08 ± 0.01
20GT	−62 ± 1	2.0 ± 0.1	0.10 ± 0.02	0.09 ± 0.01
30GT	−61 ± 1	1.7 ± 0.1	0.12 ± 0.02	0.10 ± 0.01
30GT/EmiLa	−63 ± 1	1.8 ± 0.1	0.11 ± 0.02	0.10 ± 0.01
30GT/BenmiCl	−63 ± 1	1.9 ± 0.1	0.12 ± 0.02	0.12 ± 0.01

**Table 6:** Thermo-oxidative aging factor (A_f_) of NR composites filled with ground tea waste.

NR Composite	A_f_ (-)
Unfilled NR	0.6 ± 0.1
10GT	0.8 ± 0.1
20GT	0.8 ± 0.1
30GT	0.8 ± 0.1
30GT/EmiLa	0.9 ± 0.1
30GT/BenmiCl	0.9 ± 0.1

**Table 7:** Onset decomposition temperature (T_5%_), DTG peak temperature (T_DTG_) and total mass loss (∆m) during thermal decomposition of NR composites filled with ground tea waste (SD: T_5%_ ± 1.2 °C; T_DTG_ ± 1.2 °C; ∆m ± 1.2%).

NR Composite	T_5%_ (°C)	T_DTG_ (°C)	∆m_25–600°C_ (%)	∆m_600–800°C_ (%)	Residue at 800 °C (%)
Unfilled NR	317	398	94.7	1.0	4.3
10GT	285	396	92.3	3.0	4.7
20GT	273	396	90.7	4.8	4.5
30GT	265	396	88.9	6.3	4.8
30GT/EmiLa	257	396	89.3	6.7	4.0
30GT/BenmiCl	269	398	89.4	6.9	3.7

**Table 8:** Onset decomposition temperature (T_5%_), DTG peak temperature (T_DTG_) and total mass loss (∆m) during thermal decomposition of ground tea waste (SD: T_5%_ ± 1.2 °C; T_DTG_ ± 1.2 °C; ∆m ± 1.2%).

Sample	T_5%_ (°C)	T_DTG_ (°C)	∆m_25–160°C_ (%)	∆m_160–850°C_ (%)	∆m_850–1000°C_ (%)	Residue at 1000 °C (%)
GT powder	195	339	3.7	64.2	28.3	3.8

**Table 9:** Onset decomposition temperature (T_5%_), DTG peak temperature (T_DTG_) and total mass loss (∆m) during thermal decomposition of ILs (SD: T_5%_ ± 1.2 °C; T_DTG_ ± 1.2 °C; ∆m ± 1.2%).

Sample	T_5%_ (°C)	T_DTG_ (°C)	∆m_25–600°C_ (%)	∆m_600–800°C_ (%)	Residue at 800 °C (%)
EmiLa	190	279	98.8	1.0	0.2
BenmiCl	232	313	99.4	0.4	0.2

## Data Availability

The data presented in this study are available on request from the corresponding author due to privacy.
